# PET/Graphene Nanocomposite Fibers Obtained by Dry-Jet Wet-Spinning for Conductive Textiles

**DOI:** 10.3390/polym15051245

**Published:** 2023-02-28

**Authors:** Laia León-Boigues, Araceli Flores, Marian A. Gómez-Fatou, Juan F. Vega, Gary J. Ellis, Horacio J. Salavagione

**Affiliations:** 1 Instituto de Ciencia y Tecnología de Polímeros (ICTP), CSIC, Departamento de Física de Polímeros, Elastómeros y Aplicaciones Energéticas, Juan de la Cierva 3, 28006 Madrid, Spain; 2 Instituto de Estructura de la Materia (IEM), CSIC, Departamento de Física Macromolecular, BIOPHYM, Serrano 113bis, 28006 Madrid, Spain

**Keywords:** smart textiles, mechanical stability, electrical conductivity, deformation cycles

## Abstract

The combination of polyethylene terephthalate (PET), one of the most used polymers in the textile industry, with graphene, one of the most outstanding conductive materials in recent years, represents a promising strategy for the preparation of conductive textiles. This study focuses on the preparation of mechanically stable and conductive polymer textiles and describes the preparation of PET/graphene fibers by the dry-jet wet-spinning method from nanocomposite solutions in trifluoroacetic acid. Nanoindentation results show that the addition of a small amount of graphene (2 wt.%) to the glassy PET fibers produces a significant modulus and hardness enhancement (≈10%) that can be partly attributed to the intrinsic mechanical properties of graphene but also to the promotion of crystallinity. Higher graphene loadings up to 5 wt.% are found to produce additional mechanical improvements up to ≈20% that can be merely attributed to the superior properties of the filler. Moreover, the nanocomposite fibers display an electrical conductivity percolation threshold over 2 wt.% approaching ≈0.2 S/cm for the largest graphene loading. Finally, bending tests on the nanocomposite fibers show that the good electrical conductivity can be preserved under cyclic mechanical loading.

## 1. Introduction

Electronic textiles (e-textiles) and, in particular, smart textiles, which are e-textiles capable of sensing stimuli from the environment and react accordingly, have gained great interest in recent years [1,2,3,4,5]. Nowadays, smart textiles are commonly involved in ambitious objectives related to the internet of things, robotics, healthcare, portable energy harvesters, etc. [2,6,7,8].

The preparation of such textiles requires the efficient combination of the polymer fibers with the electronic component, which has evolved from the bulky electronic devices of the late 1980s, focused on meeting specific requirements mainly for military purposes, to the smaller electronic components incorporated into fabrics and principally dedicated to sensing environmental or body parameters for healthcare. However, the real integration of electronic components into textiles requires the fabrication of devices directly on the fibers using high-performance materials that allow seamless incorporation into fabrics. Consequently, fiber-based devices are rapidly developing as an alternative and versatile platform that can offer functionality in a variety of configurations due to their peculiar geometry, aspect ratio, feature sizes, and mechanical properties [1,9]. In this respect, the combination of poly(ethylene terephthalate), PET, a polymer that occupies a very relevant place in the textile industry, with graphene is emerging as a promising tool for smart conductive fibers.

The fast-growing interest in smart textiles has oriented investigations towards the search for new electronic materials beyond metals, since the incorporation of the latter into textiles involves complex, expensive, difficult-to-scale-up and environmentally unfriendly processes. In addition, the presence of metals in the final material adds much weight and toxicity and imparts limited mechanical compliance to stress. Conducting polymers (CP) have been proposed as conductive elements in textiles due to their high solution processability, light weight and mechanical properties, which are closer to textile polymers than metals [10]. Very recently, ultrafine polyaniline fibers with good mechanical properties and acceptable electrical conductivity have been developed [11]. However, CPs also present limitations, such as lower charge transport, poor mechanical properties, instability under ambient conditions and degradation with usage due to de-doping [12]. In this sense, the fascinating electrical, mechanical and thermal properties of graphene, and its advantages over traditional metal-based technology—reduced toxicity, higher flexibility, less weight, lower processing temperature, etc.—have stimulated considerable activity in the development of functional textiles that incorporate graphene [13,14,15].

Graphene or its derivatives have been incorporated into textiles principally using coating techniques from appropriate inks [16,17,18,19,20] or by transferring CVD-grown graphene to the textile fiber surface [21,22,23]. While the latter methodology presents a broad limitation from the scalability point of view, coating approaches present some aspects to be considered. The first is related to the type of graphene or derivative employed. Due to its good solubility and supramolecular interactions with common textile fibers that are somewhat polar (PET, Nylon, cotton, etc.), graphene oxide (GO) has been preferred [20,24]. However, GO requires an additional reduction step to recover its electrical conductivity, where harsh chemical or thermal treatments can lead to the degradation, chemical modification and/or hydrolysis of the textile, eventually worsening its original mechanical properties. The use of previously reduced graphene oxide (rGO) dispersions has also been reported [17,18]. Nevertheless, rGO dispersions are less concentrated, resulting in low electrical conductivity levels and requiring several padding cycles that appear to be time-consuming and more expensive. Recently, we described an alternative approach for the preparation of nanocomposite coatings based on graphene and an elastomer, poly(styrene-b-ethylene-co-butylene-b-styrene) (SEBS) [19]. This strategy presents the important advantage of using pristine graphene (neither GO nor rGO). In addition, it produces mechanically stable and washable coatings because the elastomer confers flexibility and hydrophobicity to the conductive coating. Furthermore, nanocomposite inks prepared following this procedure can be used for coating different natural and synthetic textiles.

Beyond the initial interest, the research [25,26,27,28] on nanocomposites of PET with graphene and derivatives seems to have been reactivated in recent years [29,30,31,32,33,34,35,36]. Considering the importance of the filler/matrix interface in achieving nanocomposites with improved or new properties, graphene oxide has been the preferred graphene derivative, as, in principle, it is able to establish hydrogen bonding interactions with PET. Recent studies investigated the effect of the GO aspect ratio and surface chemistry on the mechanical properties of its nanocomposites with PET prepared by melt compounding [31,34,36]. They demonstrated that surface functionalization with trimellitic anhydride generated stronger filler/polymer interfaces with enhanced load transfer [36]. In addition, they concluded that the microstructure of the polymer, which regulates the mechanical properties, strongly depends on the filler aspect ratio [31,34]. However, the low electrical conductivity of GO limits its use in materials for electronics or smart textiles. Nanocomposites of graphene nanoplatelets (GNP) and PET with a low percolation threshold and electrical conductivity of 10 S·m^−1^ have been prepared by melt mixing, where the dispersion of GNP in the matrix is assisted by an ethylene methyl acrylate copolymer (EMA) [32]. Nevertheless, for electronic or smart textiles, these graphene/PET nanocomposites need to be processed in the form of fibers, and, to the best of our knowledge, examples are scarce [30,33,37].

According to preceding studies, the fiber morphology and mechanical and electrical properties are directly related to the choice of fiber formation process (dry or wet-jet or combined), including the type of precipitation bath [38,39,40]. Here, we report on the preparation and characterization of PET/graphene nanocomposite fibers by a dry-jet wet-spinning method using precision fluid dispensing equipment. The main challenge is to incorporate electrical conductivity into the fibers while preserving their mechanical properties. Mechanical measurements were undertaken on an isolated fiber using the most advanced nanoindentation techniques and the results are discussed as a function of graphene content. Morphological and structural studies by electron microscopy and X-ray diffraction, respectively, are correlated with mechanical performance and electrical conductivity. Finally, with regard to the potential application of PET/graphene fibers for e-textiles, the stability of electrical conductivity under cyclic bending has also been explored.

## 2. Materials and Methods

### 2.1. Chemicals 

PET (NovaPET CR pellets with intrinsic viscosity 0.8 dL·g^−1^) was kindly supplied by AITIIP (Zaragoza). Trifluoroacetic acid, TFA (CAS No.: 76-05-1) and methanol (CAS No.: 67-56-1) were supplied by Sigma-Aldrich.

**Figure 1:**
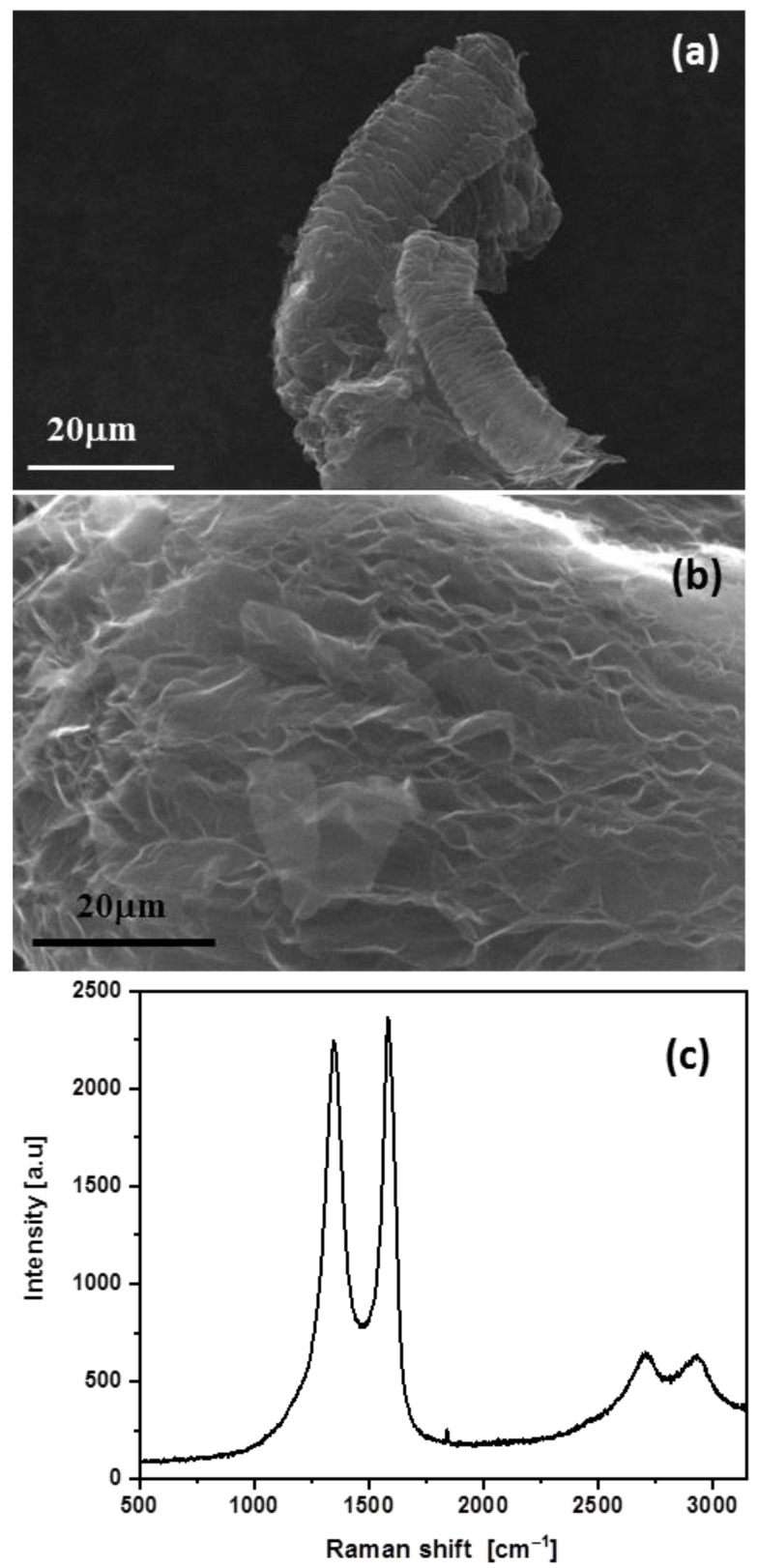
SEM images at different magnifications (**a**,**b**) and Raman spectrum (**c**) of the G employed for PET/G nanocomposite fibers.

Graphene (G) with a thickness of <3 layers, an average particle size of ~40 µm, a BET surface of 480 m^2^·g^−1^ and oxygen content (XPS) of ~2.5% was obtained from Avanzare Nanotechnology (La Rioja, Spain). The G was characterized by SEM and Raman spectroscopy (Figure 1) and the electrical conductivity measured. The morphological characterization showed the typical worm-like shape of expanded graphite and indicated the presence of layered graphene structures [41]. The stacked graphene laminates appeared wrinkled, which is typical of graphene sheets obtained by the oxidation of graphite followed by high thermal reduction [42]. The most important features in the Raman spectra, obtained using a laser excitation of 514.5 nm, were the G band appearing around 1583 cm^−1^, corresponding to the first-order scattering of the E_2g_ mode; the disorder-induced D band at 1347 cm^−1^; the second-order 2D band at around 2706 cm^−1^; and the D + G band at 2929 cm^−1^. The I_D_/I_G_ intensity ratio clearly resembled that reported for reduced graphene oxide [42]. The electrical conductivity, measured on a compressed powder pellet of G, was >100 S·cm^−1^.

### 2.2. Preparation of Nanocomposite Fibers

For the preparation of the fiber’s precursor PET/graphene inks, the filler and the polymer were separately dispersed and dissolved in TFA. A graphene dispersion with a concentration of 3 mg·mL^−1^ was prepared using a Hielscher USP400s sonication probe with a sonotrode tip diameter of 7 mm, at a working frequency of 24 kHz and amplitude of 40%, for 15 min. A polymer solution with a PET concentration of ~18 wt.% was prepared by dissolving it in TFA under magnetic stirring. Subsequently, appropriate amounts of each component were mixed under magnetic stirring and part of the solvent was allowed to evaporate at 60–68 °C in order to increase the viscosity of the mixture. Nanocomposite inks with 2, 3, 4 and 5 wt.% of graphene were studied.

Fiber spinning was conducted via the dry-jet wet-spinning approach using an ULTIMUS^TM^ precision fluid dispenser from NORDSOM EFD, coupled to a polypropylene syringe with a stainless-steel needle tip. After testing needles of different diameters, a 0.84 mm diameter was selected. The air gap between the needle and the methanol coagulation bath was ~3 cm and a pressure of 25.0 psi was applied. The fibers were dried under a vacuum at 60–65 °C for 96 h.

### 2.3. Characterization

The dispersion of graphene in the fibers was examined by scanning electron microscopy (SEM) using a Hitachi SU8000 field emission microscope (Tokyo, Japan). The nanocomposite fibers were cryofractured and images were collected at 0.8 kV, using a secondary electron and backscattered electron detector combination.

Thermogravimetric analysis (TGA) was carried out using a TA Instruments Q50 thermobalance (Waters Cromatografía, S.A., Cerdanyola del Vallès, Spain) between 50 and 800 °C at a heating rate of 10 °C·min^−1^, under an inert atmosphere (nitrogen, 60 cm^3^·min^−1^). Samples were analyzed using the TA Instruments Universal Analysis 2000 software (version 4.5A, Build 4.5.0.5).

Differential scanning calorimetry was performed in a DSC25 with a RSC90 refrigerated cooling system (TA Instruments). Samples of approximately 5 mg were placed in hermetically sealed aluminum pans. Scans were carried out from 0 to 300 °C at 10 °C·min^−1^ under a nitrogen atmosphere (flow of 50 mL·min^−1^).

**Figure 2:**
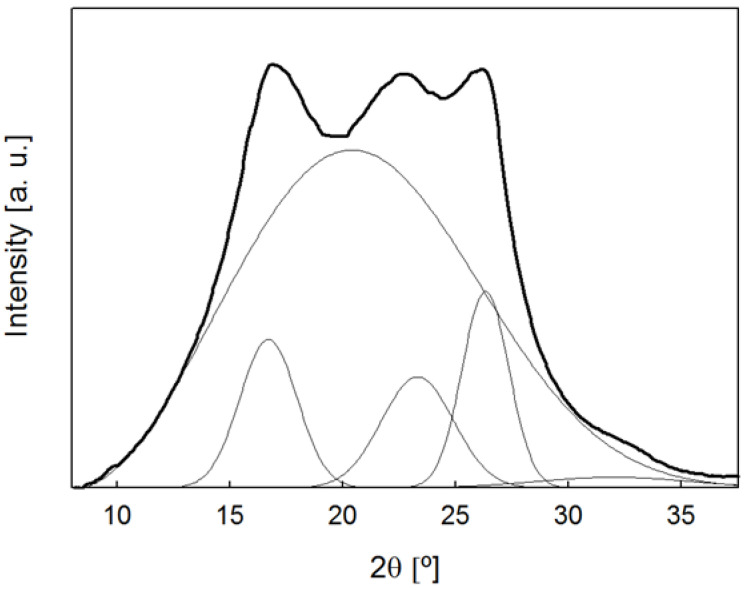
Intensity profile as a function of diffraction angle for a PET/graphene 5 wt.% fiber with 575 μm radius. The curve is separated into an amorphous halo and several crystalline peaks.

Two-dimensional wide-angle X-ray diffraction (WAXS) images were obtained with the fiber axis perpendicular to the incident beam, using a Bruker AXN diffractometer operating at 50 kV and 1 mA, with wavelength λ = 0.1542 nm. The beam size was around 100 μm × 100 μm. A photon detector was used with a resolution of 1024 × 768 pixels and 135 μm/pixel. The sample-to-detector distance was 40 mm. Background-subtracted diffraction patterns were analyzed using the FIT2D software [43]. Azimuthal scans over the main crystalline reflections showed no intensity maxima and this suggested no preferred crystal orientation. All diffraction images were integrated along the azimuthal angle to obtain intensity curves as a function of diffraction angle. As an example, Figure 2 shows the intensity profile for the PET/graphene 5 wt.% fiber. The Peakfit program (Systat Software, San Jose, CA, USA) was used to fit the intensity curves to several crystalline peaks and an amorphous halo, as shown in Figure 2. The degree of crystallinity, *X_c_*, was calculated from the ratio of the area under the crystalline peak to that of the total diffraction curve.

In order to determine the effect of the graphene loading on the electrical conductivity, the fibers were pressed into thin films and DC conductivity measurements were carried out using a four-probe setup comprising a dc low-current source (LCS-02) and a digital microvoltmeter (DMV-001) from Scientific Equipment & Services Pvt, Ltd. (Roorkee, India). For the study of electrical conductivity under cyclic bending, the electrical resistance of each type of fiber was measured in a two-contact configuration with silver paint tracks and using a low-current digital voltammeter. The fibers were subjected to a 90-degree bending regime for 3 s, and then the perturbation was released to allow the fibers to recover their original extended shape, and the electrical resistance was recorded.

Nanoindentation tests were carried out by the application of a small load (≈15 mN) on the fibers’ cross-sections. Fibers were mounted vertically using a plastic clip and subsequently embedded in epoxy resin. The cross-sections of the fibers were exposed using a microtome and the surfaces were polished using progressively finer sandpaper and finally finished with a microcloth lubricated with alumina paste. The resulting cylindrical blocks were placed on the platform of a G200 nanoindenter (KLA Tencor, Milpitas, CA, USA). A low-load head (DCM) including a Berkovich indenter was used. Experiments were carried out under dynamic testing. The load *P* was incremented exponentially with time to achieve a constant strain rate (*P’*/*P* = 0.05 s^−1^), and, at the same time, a small oscillation force was applied. A continuous measurement of the stiffness as a function of indent depth *h* was determined from the phase lag between the oscillation force and the harmonic displacement produced [44]. Finally, the storage modulus *E’* and hardness *H* could be calculated following the procedure of Oliver and Pharr and assuming elastic–viscoelastic correspondence [44,45].

Dynamic Mechanical Analysis (DMA) was performed in a Perkin Elmer DMA7 using fiber extension fixtures at room temperature (T = 20 °C) in the controlled tensile stress mode. Measurements were made in the linear viscoelastic region (LVR) by dynamic force sweeps between 100 and 3000 mN at a fixed frequency of 1 Hz. The generated dynamic strain ε as a consequence of the imposed dynamic stress σ* varied up to a maximum value of 0.35 % (ε = 0.0035), well within the LVR. The values of the complex tensile modulus E* at 1 Hz were obtained from the slope of the plot of the imposed applied tensile dynamic stress versus the produced tensile dynamic strain.

## 3. Results

**Figure 3:**
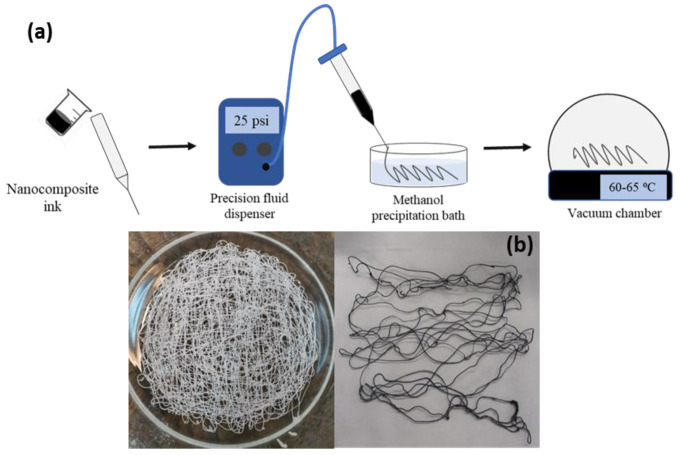
(**a**) Scheme of dry–jet wet–spinning method using a precision fluid dispenser and manually creating the fibers. (**b**) Photograph of PET (left) and PET/graphene fibers with a loading of 4 wt.% prepared by dry–jet wet spinning.

As the real integration of electronic components into textiles requires the incorporation of nanoscale conductive fillers directly in the fibers, the optimization of the spinning conditions (spinning solution solvent, coagulation bath and temperature, spinning approach, etc.) is fundamental to obtain fibers with balanced mechanical and electrical properties. We initially approached wet spinning from TFA solutions and coagulation in methanol and water baths, but either no fiber formation or fibers with poor mechanical stability were produced. TFA has been suggested as a solvent that meets the solubility–spinnability requirements for the production of PET fibers [46,47], and methanol is a good solvent for polymer precipitation. On the other hand, it has been demonstrated that, using the dry-jet wet spinning of graphene and its derivatives, the introduction of an air gap between the tip and the coagulation bath resulted in fibers with superior mechanical properties [38,48]. Therefore, we explored the dry-jet wet spinning of PET/graphene solutions using a precision fluid dispenser and producing the fibers manually (see Figure 3a). Figure 3b shows examples of the samples obtained with this approach.

**Figure 4:**
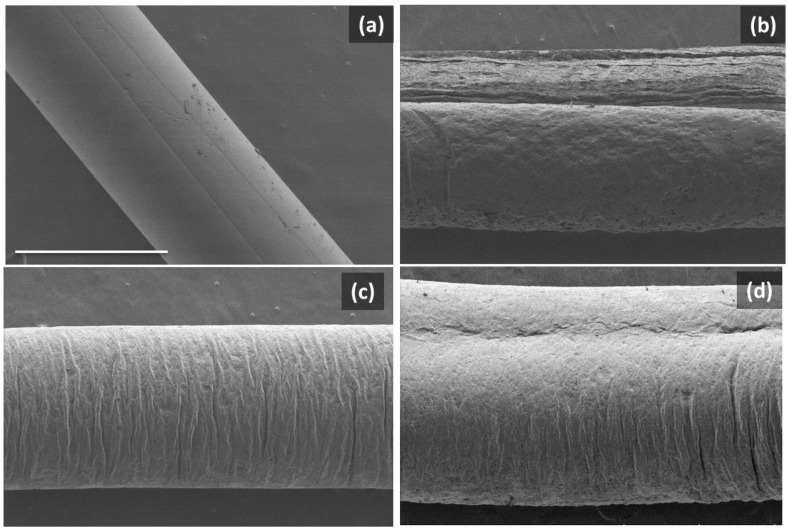
SEM images of the surfaces of dry-jet-wet-spinning-produced fibers: (**a**) PET and PET/graphene with loadings of (**b**) 2 wt.%, (**c**) 4 wt.% and (**d**) 5 wt.%. Scale bar in (**a**) applies to all images and corresponds to 500 microns.

**Figure 5:**
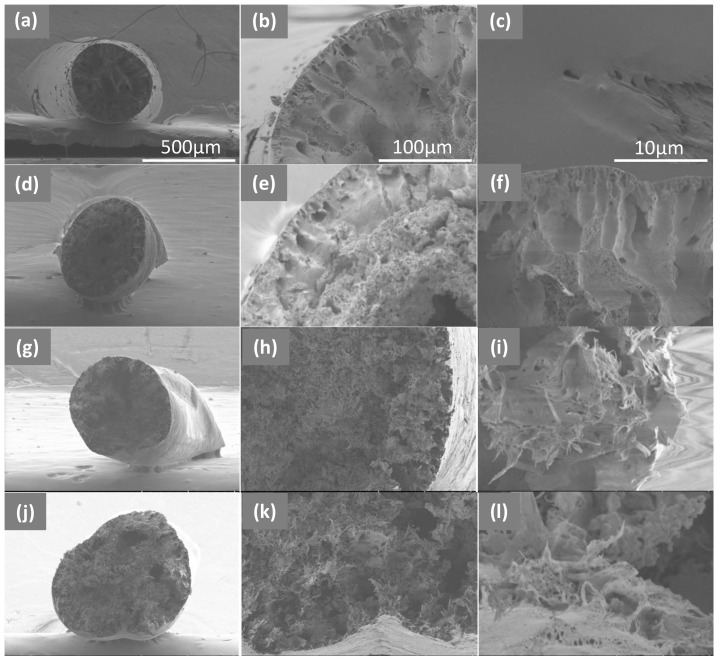
SEM images at different magnifications of the cross-sections of cryofractured samples of PET (**a**–**c**) and PET/graphene with loadings of 2 wt.% (**d**–**f**), 4 wt.% (**g**–**i**) and 5 wt.% (**j**–**l**). Scale bar in (**a**–**c**) applies to the images in the same column.

The morphology of all PET/graphene fibers was evaluated by SEM (Figure 4 and Figure 5). Images comparing the surfaces of nanocomposite fibers with that of a pristine PET fiber are shown in Figure 4. In all cases, the fibers presented a uniform size as the diameter did not change along the fiber axis. In addition, it was found that the initially smooth side-walls of the PET fiber changed to a rougher surface for the nanocomposites, the roughness increasing with the graphene content.

Fibers were studied in depth by analyzing the cross-sections on cryofractured samples. From Figure 5, it can be seen that the presence of graphene induces a slight change in fiber shape. Moreover, all nanocomposite fibers present a well-packed morphology where graphene is homogeneously distributed throughout the area of the fiber, with no aggregates, suggesting good dispersion. For the sample with the lowest graphene loading, the cross-section suggests that graphene may be preferentially located in the fiber center. As a consequence of the spinning process and the selected coagulation bath, during the initial stages of the spinning process, graphene may tend to occupy the inner parts of the fiber. As the graphene concentration increases, the filler distribution could expand in the radial direction towards the outer surface. Regarding fiber performance, it is expected that both the homogeneous distribution of graphene and better morphological packing arising from the dry-jet wet-spinning process used will influence the electrical and mechanical properties [49], as discussed below. In addition, some voids with an irregular distribution were observed in all samples, which could have an influence on the mechanical properties.

**Figure 6:**
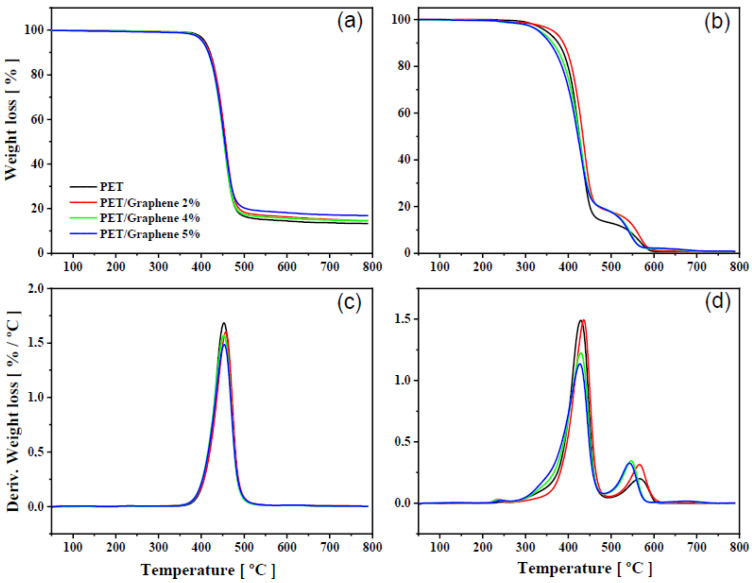
TGA and dTGA curves for PET and PET/graphene nanocomposites with different graphene loadings under nitrogen (**a**,**c**) and air (**b**,**d**) atmospheres. Heating scan = 5 °C·min^−1^.

The thermal stability of PET/graphene nanocomposites under nitrogen and air atmospheres was investigated and results are shown in Figure 6. All TGA curves display a flat profile at temperatures below 350 °C, indicating total elimination of the TFA solvent during the drying process. The TGA curves of pure PET and the nanocomposites with different graphene content under nitrogen gas conditions suggest that the filler has a marginal effect on the thermal stability of the polymer. A single-stage mass loss process was observed between 350 and 510 °C, associated with the decomposition of the polymer. This result is somewhat expected as relatively high graphene loadings were used in this work. Thus, the initial thermal stabilization at low graphene loadings, where graphene acts as a barrier that can hinder the diffusion of the degradation products, slowing down the decomposition process, is counterbalanced at higher loadings by the accumulation of graphene, reducing the influence of the filler on the thermal stability [50,51]. This effect is confirmed by experiments in an air atmosphere (Figure 6b,d). In this case, the thermal degradation of PET and its composites occurs in two steps; the former is associated with the main polymer chain breakdown and formation of char, and the second is a retarded degradation process due to the formation of more fragmented oxidative products [52]. Regarding the nanocomposites, the sample with lowest graphene loading (2 wt.%) shows slight thermal stabilization of the first process as the onset temperature and the temperature of the maximum degradation rate are displaced to higher values, while the second process is quite similar to those observed in PET. However, in samples with higher graphene content, the first process resembles that of PET and the second process is accelerated and occurs at lower temperatures. The thermal stability of PET nanocomposites with carbon nanofillers in an air atmosphere displays dissimilar results as this process is shifted to both lower and higher temperatures [27,53,54,55]. Particularly interesting are the cases of nanocomposites prepared by the melt compounding of PET and exfoliated graphite (EG), where shifts to higher temperatures of 42 and 32 °C for the first and second process, respectively, are reported for samples with high graphene loadings of up to 7 wt.% [27]. Although solution mixing as reported in our study is expected to render better filler/polymer interfaces, melt compounding can induce some preferential filler orientation in the matrix that can help in improving some properties [56]. In this case, such a substantial enhancement in the thermal stability under air can be attributed to a barrier effect to the volatile decomposed products of graphene sheets that are well dispersed and oriented in the PET matrix.

**Figure 7:**
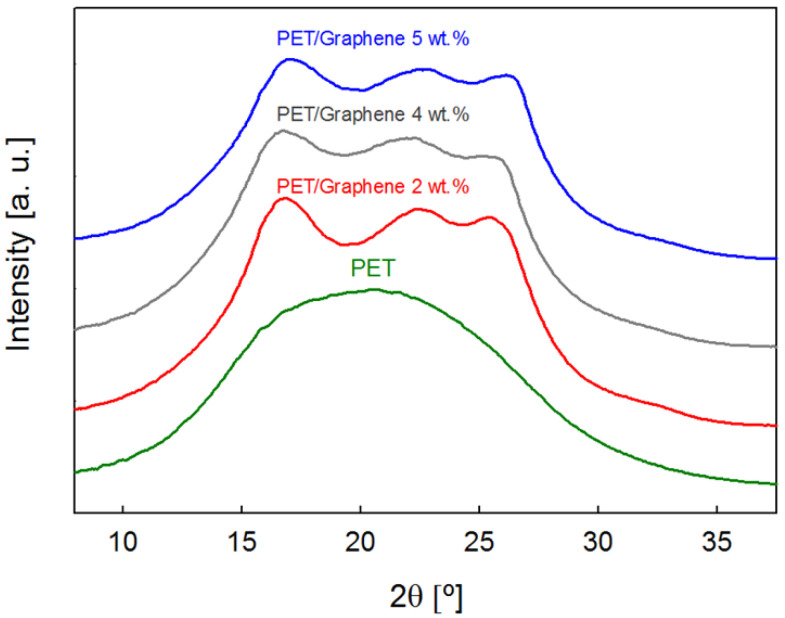
Scattered intensity as a function of diffraction angle for pristine PET and PET/graphene fibers with different filler content. Curves are shifted for the sake of clarity.

Most interesting is the influence of graphene on the PET fiber nanostructure. The WAXS patterns of pristine PET fibers exhibit a clear broad halo characteristic of an amorphous material (see Figure 7). In contrast, the diffraction images of all fibers exhibit crystalline maxima (Figure 7), and analysis of the crystallinity levels for fibers with the same diameter size yields *X_c_* = 23–29%. Table 1 shows that the crystallinity levels do not follow a clear trend with the quantity of graphene.

**Figure 8:**
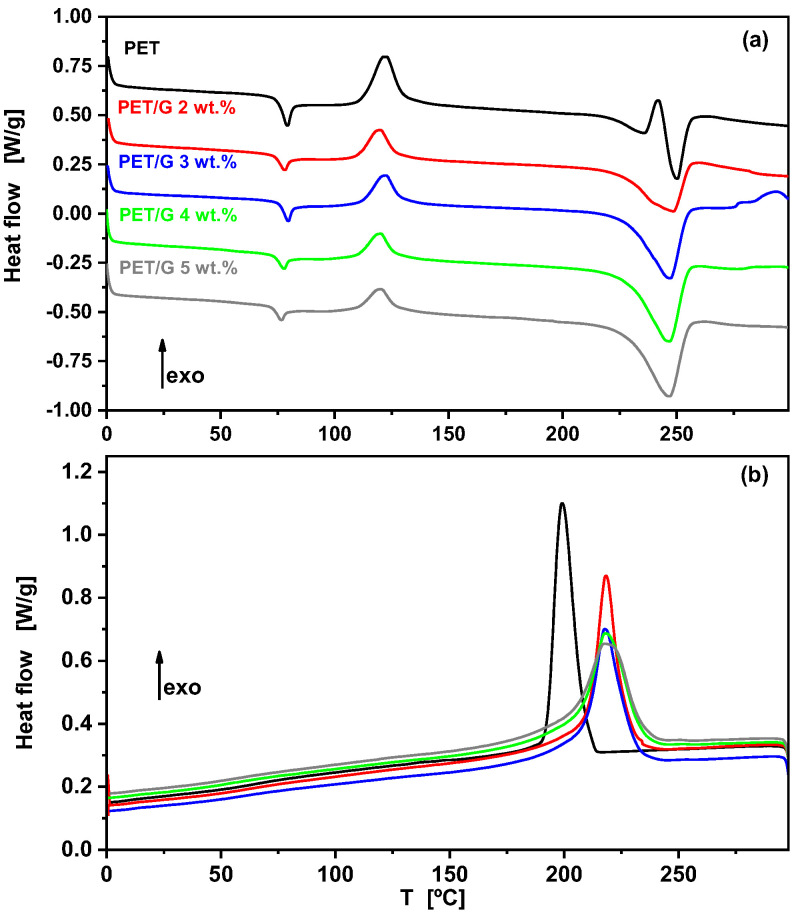
DSC heating scan for PET fibers (**a**) and subsequent cooling scan (**b**).

The fibers were also analyzed by DSC and the curves of the first heating scan are shown in Figure 8a. On heating, after the glass transition at around 70 °C, all fibers presented a cold crystallization process at around 122 °C. However, the crystallization enthalpy of this process was much smaller in the fibers with graphene as compared with pure PET fibers, corroborating the presence of initial crystallinity due to the nucleating effect of graphene, as observed in the X-ray results described above. This nucleating effect was clearly detected in the subsequent cooling scans from the melt after eliminating the thermal history of the fibers (Figure 8b). The crystallization temperatures of the nanocomposites were ~20 °C higher than that for the neat polymer. A similar increase was observed for nanocomposites of PET with different grades of graphene oxide, prepared by melt mixing [33,34] or electrospinning [30].

**Figure 9:**
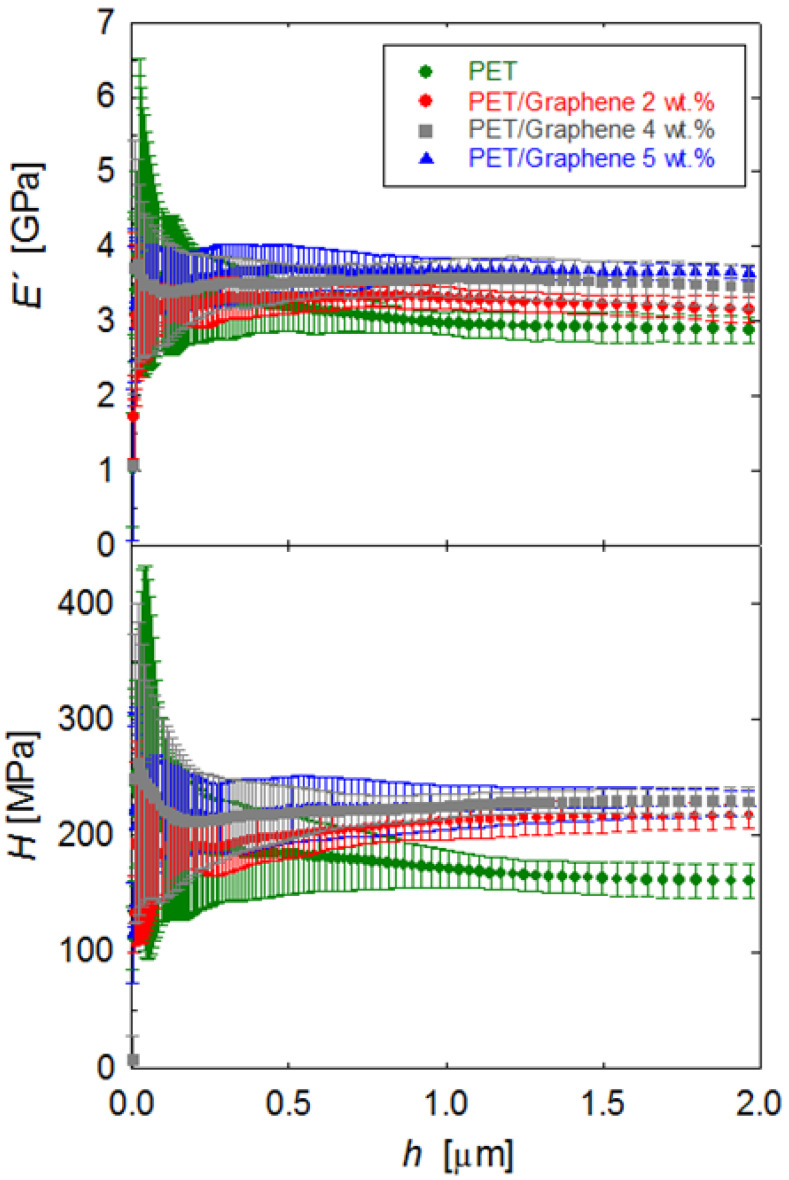
*E´* and *H* behavior as a function of indent displacement, for PET and PET/graphene fibers.

The structural differences described above upon the addition of graphene can be correlated to variations in the mechanical behavior. Nanoindentation testing was carried out on the cross-sections of all PET/graphene and pristine PET fibers, and Figure 9 shows the evolution of *E´* and *H* as a function of indenter displacement into the surface. Error bars arise from the statistical analysis of indentations produced at different locations to cover the whole fiber cross-sectional area. The high noise at shallow depths *h* < 500 nm can be associated with the tip–sample interaction and the surface roughness of each particular fiber [50]. These effects gradually lose relevance as the indenter progresses towards the bulk, and, in all cases, the *E´* and *H* values eventually remain constant with an indent depth beyond *h* ≈ 1 μm. Moreover, also beyond this point, the standard deviation in the data is quite similar in all samples. Hence, it seems that the addition of graphene does not introduce mechanical heterogeneities at the micrometer scale, and *E´* and *H* for *h* > 1 μm represent average values for each material. It is worth recalling that the deformation volume typically extends up to ≈10 times the indent depth for the plastic field and ≈20 times for elastic deformation.

**Figure 10:**
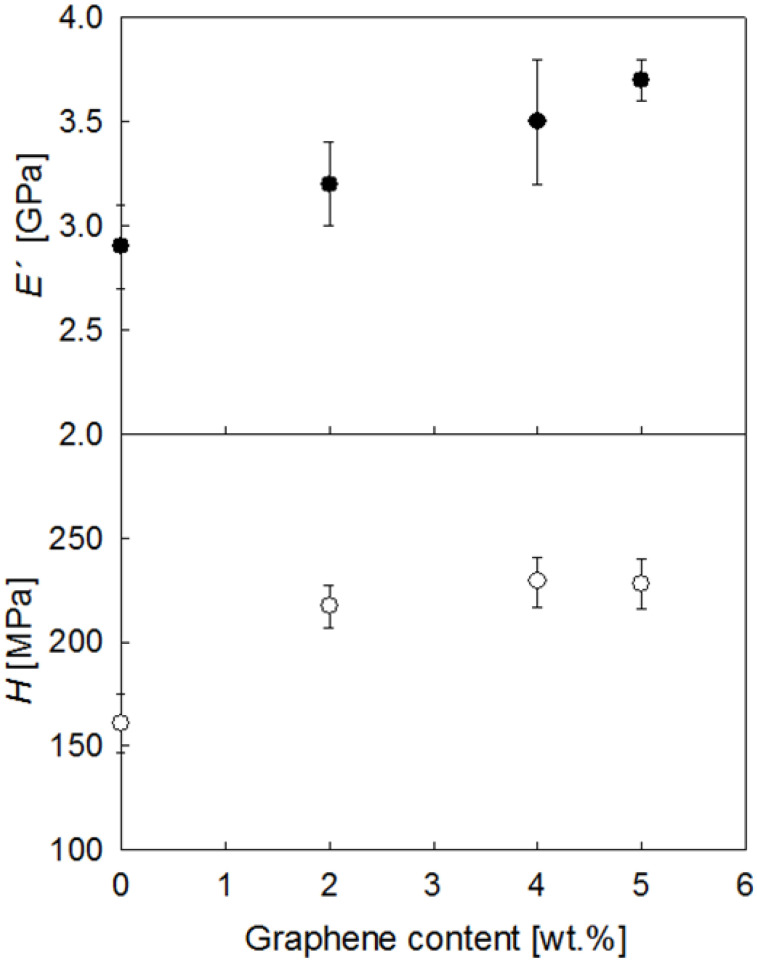
Storage modulus and hardness as a function of graphene content for PET and PET/graphene fibers.

Figure 10 shows the plot of the *E´* and *H* values taken at *h* = 2 μm as a function of graphene content for all materials investigated. In the first place, the *E´* and *H* values for the pristine PET fiber are in agreement with earlier values in other PET systems [57,58]. Moreover, it is seen that the addition of graphene enhances the modulus and hardness values of the PET fiber. For the largest quantities of graphene, *E´* and *H* improvements of 28% and 42%, respectively, are found. The initial *E´* and *H* improvement with the incorporation of the smallest quantity of graphene (2 wt.%) can be associated with both the development of matrix crystallinity (from *X_c_* = 0 to *X_c_* = 28%, see Table 1) and the intrinsic superior mechanical properties of graphene. The addition of larger graphene quantities is found to produce further mechanical enhancements, and this can be solely attributed to the filler, taking into account that the matrix crystallinity remains almost constant (see Table 1).

The dynamic tensile measurements were found to be in agreement with the indentation results at low graphene loadings. The longitudinal strength of nanocomposite fibers with 2 wt.% of graphene was found to be around 33% higher than that of the non-reinforced PET (E* = 4.0 ± 0.3 GPa), which is probably due to the development of crystallinity and the superior properties of the graphene filler. The longitudinal strength of the 4 wt.% graphene fiber (E* = 5.0 ± 0.3 GPa) only increased by around ≈25% compared to non-reinforced PET and is slightly lower than that for the sample with a loading of 2 wt.%. This may be attributed to heterogeneities along the sample and a decrease in interfacial filler/polymer interactions due to the formation of graphene aggregates. In this respect, it is also worth commenting that the modulus values could not be determined for PET/graphene fibers with the highest graphene content (5 wt.%), as a consequence of fiber breaking events during sample fixation. Such observations suggest that high graphene loading promotes fiber fragility, and this could also be correlated with the enhanced surface roughness observed by electron microscopy with increasing graphene content (see Figure 4). In addition, the generation of voids in the fiber structure should not be overlooked as it may also influence the mechanical performance of the fibers. Despite this, the results clearly demonstrated that graphene addition improved the fiber strength.

One of the main goals of this work was to prepare textile fibers that presented electrical conductivity, while preserving the good mechanical properties of PET, dedicated to flexible and wearable electronics (e-textiles) such as sensors, wearable heating devices and healthcare monitoring devices, among others. Table 2 lists the conductivity values for fibers containing different graphene loadings. It can be seen that good conductivity values, in the range of 0.02 S/cm to 0.18 S/cm, are obtained with loadings over 3 wt.%. As the sample with 2 wt.% shows no measurable conductivity in the range of detection of the equipment, it can be assumed that the percolation threshold lies between 2 and 3 wt.%. As expected, for samples with loadings higher than 3 wt.%, the conductivity increases with the graphene loading. The conductivity values achieved in this study fall within the range required for the aforementioned applications [59] and are higher than the best results reported so far for conducting fibers with similar compositions. In order to appraise the values obtained in this work, Table 3 establishes a comparison with the data in the literature for conducting fibers of several families of nanocomposites [11,26,27,28,31,60,61,62,63,64,65,66,67,68,69,70,71,72,73]. As can be seen from Table 3, better conductivity is only achieved for fibers containing 30 wt.% of polyaniline (PANI) as the conducting element (entry 6 in Table 3). The mechanical properties of PANI are somewhat poor, and, for this reason, fibers of its nanocomposites with polyacrylonitrile (PAN) or polyethylene oxide (PEO) have been investigated. For these nanocomposites, conductivity values similar to those presented in this study were reported (see entries 3 and 9 in Table 3), although with a much higher loading of PANI. Focusing on the specific case of the nanocomposites addressed in this study, the conductivity values are much higher than those reported thus far for PET/G fibers (entry 19 in Table 3). Further, they are higher than the values for hot-pressed PET/G nanocomposite films (entries 13–17 in Table 3), except for the case reported in reference [28] (entry 18 in Table 3).

**Figure 11:**
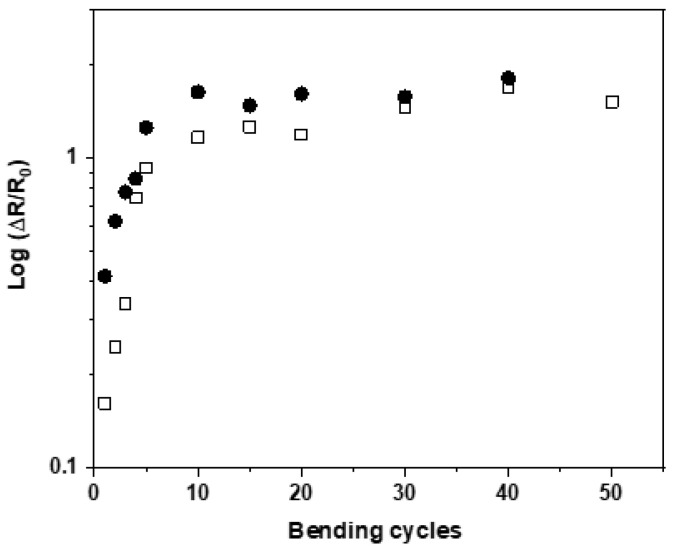
Variation in the resistance of PET/G fibers with different graphene loadings with the number of bending cycles (open squares and full circles correspond to loadings of 4 wt.% and 5 wt.%, respectively).

Regarding e-textile applications, it is very important that the conducting fibers preserve the conductivity under mechanical deformation, recovering their initial values after the perturbation is released. Thus, the variation in the resistance of the nanocomposite fibers under repetitive bending/release cycles was investigated. Figure 11 shows the variation in the resistance of the fibers containing 4 wt.% (open squares) and 5 wt.% (full circles) of graphene subjected to fifty bending cycles. It can be seen that despite an initial increase in the electrical resistance, the values stabilize, showing only small variations from cycle 5 to 50. The significant increase in electrical resistance during the first bending cycle could be due to some rearrangement of the filler within the polymer matrix.

## 4. Conclusions

The optimized dry-jet wet-spinning preparation of PET/graphene nanocomposite fibers with balanced mechanical and electrical properties using precision fluid dispensing equipment is presented. TFA and methanol were selected as the solvent and precipitation bath, respectively.

Fibers with good thermal stability and a homogeneous dispersion of graphene were developed. Graphene was found to induce crystallinity in the fibers, as observed by X-ray diffraction and DSC. Such structural changes, together with the intrinsic superior properties of graphene, produce a modulus and hardness improvement that reaches 28% and 42% for the largest quantity of graphene (5 wt.%). Finally, the nanocomposite fibers exhibit good electrical conductivity stability under cyclic bending. Hence, the PET/graphene fibers appear to be promising candidates for applications in e-textiles.

## Figures and Tables

**Table 1:** Degree of crystallinity of all PET/graphene and pristine PET fibers with ≈550 μm diameter size, as determined from WAXS analysis.

Fiber Type	*X_c_*
PET	0
PET/Graphene 2 wt.%	0.29
PET/Graphene 4 wt.%	0.23
PET/Graphene 5 wt.%	0.28

**Table 2:** Conductivity values for fibers containing different graphene loadings.

Fiber Type	Conductivity (S/cm)
PET/Graphene 2 wt.%	-
PET/Graphene 3 wt.%	0.020 ± 0.003
PET/Graphene 4 wt.%	0.103 ± 0.032
PET/Graphene 5 wt.%	0.181 ± 0.010

**Table 3:** Comparison of the electrical conductivity of different conductive fibers.

Entry	Fiber Type	Conducting Component Loading (wt.%)	Nanocomposite Form	Electrical Conductivity (S/cm)	Reference
1	PANI	100	Fibers by wet spinning	~5 × 10^−4^	[11]
2	PANI	100	Fibers by electrospinning	0.03	[60]
3	PEO/PANI/	98.5–99.9	Fibers by electrospinning	~10^−4^–10^−3^	[61]
4	PEO/PANI	93	Fibers by electrospinning	0.144	[62]
5	PEO/PANI	10–40	Fibers by electrospinning	~10^−6^–10^−3^	[63]
6	PEO/PANI		Fibers by electrospinning	<3.1 × 10^−11^	[64]
7	Silk fibroin/PANI	2.5–30	Fibers by electrospinning	Up to 0.5	[65]
8	PVAc/PANI	50–66	Fibers by electrospinning	2.5 × 10^−5^–3.6 × 10^−5^	[66]
9	PAN/PANI	1–3	Fibers by electrospinning	~7 × 10^−3^–2.8 10^−2^	[67]
10	PAN/PANI	10–30	Fibers by electrospinning	~10^−5^–0.1	[68]
11	PAN/PANI	16	In situ aniline polymerization on PAN fibers	1.8 × 10^−4^	[69]
12	PAN/PANI	25–43	Fibers by electrospinning	<7 × 10^−9^	[70]
13	PET/PANI	1–9	PANI coating on PET mats	~1.7 × 10^−3^–10^−2^	[71]
14	PET/G	0.1–7	Hot-pressed films from melt-compounded nanocomposites	Up to 10^−6^	[27]
15	PET/G	0.1–0.4	Hot-pressed films from injection-molded nanocomposites	Up to 10^−4^	[26]
16	PET/G	0.5–2	Hot-pressed films from melt-compounded nanocomposites	~10^−12^–10^−8^	[31]
17	PET/G	1–12	Hot-pressed films from melt-compounded nanocomposites	~10^−13^–10^−7^	[36]
18	PET/G	3	Hot-pressed films from ball-milling nanocomposites	~10^−2^	[73]
19	PET/G	0.5–3	Hot-pressed films from melt-compounded nanocomposites	~10^−11^–1	[28]
20	PET/G	0.5–4	Fibers by melt spinning from nanocomposites prepared by in situ polymerization	1.75 × 10^−9^–1.5 × 10^−8^	[73]
21	PET/G	2–5	Fibers by dry-wet jet spinning	Up to 0.18	This work

## Data Availability

Not applicable.

## References

[1] Yan W., Page A., Nguyen-Dang T., Qu Y., Sordo F., Wei L., Sorin F. (2019). Advanced Multimaterial Electronic and Optoelectronic Fibers and Textiles. Adv. Mater..

[2] Chen G., Li Y., Bick M., Chen J. (2020). Smart Textiles for Electricity Generation. Chem. Rev..

[3] Allison L., Hoxie S., Andrew T.L. (2017). Towards Seamlessly-Integrated Textile Electronics: Methods to Coat Fabrics and Fibers with Conducting Polymers for Electronic Applications. Chem. Commun..

[4] Shi J., Liu S., Zhang L., Yang B., Shu L., Yang Y., Ren M., Wang Y., Chen J., Chen W. (2020). Smart Textile-Integrated Microelectronic Systems for Wearable Applications. Adv. Mater..

[5] Choudhry N.A., Arnold L., Rasheed A., Khan I.A., Wang L. (2021). Textronics—A Review of Textile-Based Wearable Electronics. Adv. Eng. Mater..

[6] Ruckdashel R.R., Venkataraman D., Park J.H. (2021). Smart Textiles: A Toolkit to Fashion the Future. J. Appl. Phys..

[7] Gao Y., Cho J.H., Ryu J., Choi S. (2020). A Scalable Yarn-Based Biobattery for Biochemical Energy Harvesting in Smart Textiles. Nano Energy.

[8] Esfahani M.I.M., Ehrmann A., Nguyen T.A., Nguyen Tri P.B.T. (2021). Chapter 6-Smart Textiles in Healthcare: A Summary of History, Types, Applications, Challenges, and Future Trends. Micro and Nano Technologies.

[9] Ma W., Zhang Y., Pan S., Cheng Y., Shao Z., Xiang H., Chen G., Zhu L., Weng W., Bai H. (2021). Smart Fibers for Energy Conversion and Storage. Chem. Soc. Rev..

[10] Grancarić A.M., Jerković I., Koncar V., Cochrane C., Kelly F.M., Soulat D., Legrand X. (2017). Conductive Polymers for Smart Textile Applications. J. Ind. Text..

[11] Fang B., Yan J., Chang D., Piao J., Ma K.M., Gu Q., Gao P., Chai Y., Tao X. (2022). Scalable Production of Ultrafine Polyaniline Fibres for Tactile Organic Electrochemical Transistors. Nat. Commun..

[12] Wang B., Facchetti A. (2019). Mechanically Flexible Conductors for Stretchable and Wearable E-Skin and E-Textile Devices. Adv. Mater..

[13] Dubal D.P., Chodankar N.R., Kim D.H., Gomez-Romero P. (2018). Towards Flexible Solid-State Supercapacitors for Smart and Wearable Electronics. Chem. Soc. Rev..

[14] Wang C., Xia K., Wang H., Liang X., Yin Z., Zhang Y. (2019). Advanced Carbon for Flexible and Wearable Electronics. Adv. Mater..

[15] Torrisi F., Carey T. (2018). Graphene, Related Two-Dimensional Crystals and Hybrid Systems for Printed and Wearable Electronics. Nano Today.

[16] Afroj S., Tan S., Abdelkader A.M., Novoselov K.S., Karim N. (2020). Highly Conductive, Scalable, and Machine Washable Graphene-Based E-Textiles for Multifunctional Wearable Electronic Applications. Adv. Funct. Mater..

[17] Afroj S., Karim N., Wang Z., Tan S., He P., Holwill M., Ghazaryan D., Fernando A., Novoselov K.S. (2019). Engineering Graphene Flakes for Wearable Textile Sensors via Highly Scalable and Ultrafast Yarn Dyeing Technique. ACS Nano.

[18] Karim N., Afroj S., Tan S., He P., Fernando A., Carr C., Novoselov K.S. (2017). Scalable Production of Graphene-Based Wearable E-Textiles. ACS Nano.

[19] Salavagione H.J., Shuttleworth P.S., Fernández-Blázquez J.P., Ellis G.J., Gómez-Fatou M.A. (2020). Scalable Graphene-Based Nanocomposite Coatings for Flexible and Washable Conductive Textiles. Carbon N. Y..

[20] Pan Q., Shim E., Pourdeyhimi B., Gao W. (2017). Nylon-Graphene Composite Nonwovens as Monolithic Conductive or Capacitive Fabrics. ACS Appl. Mater. Interfaces.

[21] Neves A.I.S., Rodrigues D.P., De Sanctis A., Alonso E.T., Pereira M.S., Amaral V.S., Melo L.V., Russo S., De Schrijver I., Alves H. (2017). Towards Conductive Textiles: Coating Polymeric Fibres with Graphene. Sci. Rep..

[22] Torres Alonso E., Rodrigues D.P., Khetani M., Shin D.-W., De Sanctis A., Joulie H., de Schrijver I., Baldycheva A., Alves H., Neves A.I.S. (2018). Graphene Electronic Fibres with Touch-Sensing and Light-Emitting Functionalities for Smart Textiles. NPJ Flex. Electron..

[23] Neves A.I.S., Bointon T.H., Melo L.V., Russo S., de Schrijver I., Craciun M.F., Alves H. (2015). Transparent Conductive Graphene Textile Fibers. Sci. Rep..

[24] Ren J., Wang C., Zhang X., Carey T., Chen K., Yin Y., Torrisi F. (2017). Environmentally-Friendly Conductive Cotton Fabric as Flexible Strain Sensor Based on Hot Press Reduced Graphene Oxide. Carbon N. Y..

[25] Shim S.H., Kim K.T., Lee J.U., Jo W.H. (2012). Facile Method to Functionalize Graphene Oxide and Its Application to Poly(Ethylene Terephthalate)/Graphene Composite. ACS Appl. Mater. Interfaces.

[26] Paszkiewicz S., Szymczyk A., Špitalský Z., Soccio M., Mosnáček J., Ezquerra T.A., Rosłaniec Z. (2012). Electrical Conductivity of Poly(Ethylene Terephthalate)/Expanded Graphite Nanocomposites Prepared by in Situ Polymerization. J. Polym. Sci. Part B Polym. Phys..

[27] Li M., Jeong Y.G. (2011). Poly(Ethylene Terephthalate)/Exfoliated Graphite Nanocomposites with Improved Thermal Stability, Mechanical and Electrical Properties. Compos. Part A Appl. Sci. Manuf..

[28] Zhang H.-B., Zheng W.-G., Yan Q., Yang Y., Wang J.-W., Lu Z.-H., Ji G.-Y., Yu Z.-Z. (2010). Electrically Conductive Polyethylene Terephthalate/Graphene Nanocomposites Prepared by Melt Compounding. Polymer.

[29] de Souza Z.S.B., Pinto G.M., da Silva G.C., Demarquette N.R., Fechine G.J.M., Sobrinho M.A.M. (2021). Interface Adjustment between Poly(Ethylene Terephthalate) and Graphene Oxide in Order to Enhance Mechanical and Thermal Properties of Nanocomposites. Polym. Eng. Sci..

[30] Selatile K., Ray S.S., Ojijo V., Sadiku R.E. (2021). Morphological, Thermal, and Mechanical Properties of Electrospun Recycled Poly(Ethylene Terephthalate)/Graphene Oxide Composite Nanofiber Membranes. ACS Omega.

[31] Aoyama S., Ismail I., Park Y.T., Macosko C.W., Ougizawa T. (2020). PET/Graphene Compatibilization for Different Aspect Ratio Graphenes via Trimellitic Anhydride Functionalization. ACS Omega.

[32] Ozdemir E., Arenas D.R., Kelly N.L., Hanna J.V., van Rijswijk B., Degirmenci V., McNally T. (2020). Ethylene Methyl Acrylate Copolymer (EMA) Assisted Dispersion of Few-Layer Graphene Nanoplatelets (GNP) in Poly(Ethylene Terephthalate) (PET). Polymer.

[33] Yu W., Zhang X., Gao X., Liu H., Zhang X. (2020). Fabrication of High-Strength PET Fibers Modified with Graphene Oxide of Varying Lateral Size. J. Mater. Sci..

[34] Aoyama S., Ismail I., Park Y.T., Macosko C.W., Ougizawa T. (2019). Higher-Order Structure in Amorphous Poly(Ethylene Terephthalate)/Graphene Nanocomposites and Its Correlation with Bulk Mechanical Properties. ACS Omega.

[35] Yang B., Chen J., Su L.F., Miao J.B., Chen P., Qian J.S., Xia R., Shi Y. (2019). Melt Crystallization and Thermal Properties of Graphene Platelets (GNPs) Modified Recycled Polyethylene Terephthalate (RPET) Composites: The Filler Network Analysis. Polym. Test..

[36] Aoyama S., Ismail I., Park Y.T., Yoshida Y., Macosko C.W., Ougizawa T. (2018). Polyethylene Terephthalate/Trimellitic Anhydride Modified Graphene Nanocomposites. ACS Appl. Nano Mater..

[37] Khan U., Young K., O’Neill A., Coleman J.N. (2012). High Strength Composite Fibres from Polyester Filled with Nanotubes and Graphene. J. Mater. Chem..

[38] Seyedin S., Romano M.S., Minett A.I., Razal J.M. (2015). Towards the Knittability of Graphene Oxide Fibres. Sci. Rep..

[39] Um I.C., Kweon H., Lee K.G., Ihm D.W., Lee J.-H., Park Y.H. (2004). Wet Spinning of Silk Polymer: I. Effect of Coagulation Conditions on the Morphological Feature of Filament. Int. J. Biol. Macromol..

[40] Wu G., Cuculo J.A. (1995). Preparation of High Performance PET Fiber by Solution Spinning Technique. J. Appl. Polym. Sci..

[41] Xie X., Zhou Y., Huang K. (2019). Advances in Microwave-Assisted Production of Reduced Graphene Oxide. Front. Chem..

[42] Stankovich S., Dikin D.A., Piner R.D., Kohlhaas K.A., Kleinhammes A., Jia Y., Wu Y., Nguyen S.T., Ruoff R.S. (2007). Synthesis of Graphene-Based Nanosheets via Chemical Reduction of Exfoliated Graphite Oxide. Carbon N. Y..

[43] Hammersley A.P., Svensson S.O., Thompson A., Graafsma H., Kvick Å., Moy J.P. (1995). Calibration and Correction of Distortions in Two-Dimensional Detector Systems. Rev. Sci. Instrum..

[44] Herbert E.G., Oliver W.C., Pharr G.M. (2008). Nanoindentation and the Dynamic Characterization of Viscoelastic Solids. J. Phys. D Appl. Phys..

[45] Oliver W.C., Pharr G.M. (1992). An Improved Technique for Determining Hardness and Elastic Modulus Using Load and Displacement Sensing Indentation Experiments. J. Mater. Res..

[46] Mahalingam S., Raimi-Abraham B.T., Craig D.Q.M., Edirisinghe M. (2015). Solubility-Spinnability Map and Model for the Preparation of Fibres of Polyethylene (Terephthalate) Using Gyration and Pressure. Chem. Eng. J..

[47] Jafari S., Hosseini Salekdeh S.S., Solouk A., Yousefzadeh M. (2020). Electrospun Polyethylene Terephthalate (PET) Nanofibrous Conduit for Biomedical Application. Polym. Adv. Technol..

[48] Xiang C., Behabtu N., Liu Y., Chae H.G., Young C.C., Genorio B., Tsentalovich D.E., Zhang C., Kosynkin D.V., Lomeda J.R. (2013). Graphene Nanoribbons as an Advanced Precursor for Making Carbon Fiber. ACS Nano.

[49] Oksuz M., Erbil H.Y. (2018). Wet-Spun Graphene Filaments: Effect of Temperature of Coagulation Bath and Type of Reducing Agents on Mechanical & Electrical Properties. RSC Adv..

[50] Quiles-Díaz S., Enrique-Jimenez P., Papageorgiou D.G., Ania F., Flores A., Kinloch I.A., Gómez-Fatou M.A., Young R.J., Salavagione H.J. (2017). Influence of the Chemical Functionalization of Graphene on the Properties of Polypropylene-Based Nanocomposites. Compos. Part A Appl. Sci. Manuf..

[51] Salavagione H.J., Quiles-Díaz S., Enrique-Jimenez P., Martínez G., Ania F., Flores A., Gómez-Fatou M.A. (2016). Development of Advanced Elastomeric Conductive Nanocomposites by Selective Chemical Affinity of Modified Graphene. Macromolecules.

[52] Martín-Gullón I., Esperanza M., Font R. (2001). Kinetic Model for the Pyrolysis and Combustion of Poly-(Ethylene Terephthalate) (PET). J. Anal. Appl. Pyrolysis.

[53] Seyyed Monfared Zanjani J., Saner Okan B., Menceloglu Y. (2016). Manufacturing of Multilayer Graphene Oxide/Poly(Ethylene Terephthalate) Nanocomposites with Tunable Crystallinity, Chain Orientations and Thermal Transitions. Mater. Chem. Phys..

[54] Santoro G., Gómez M.A., Marco C., Ellis G. (2010). A Solvent-Free Dispersion Method for the Preparation of PET/MWCNT Composites. Macromol. Mater. Eng..

[55] Yoo H.J., Jung Y.C., Cho J.W. (2008). Effect of Interaction between Poly(Ethylene Terephthalate) and Carbon Nanotubes on the Morphology and Properties of Their Nanocomposites. J. Polym. Sci. Part B Polym. Phys..

[56] López-González M., Flores A., Marra F., Ellis G., Gómez-Fatou M., Salavagione H.J. (2020). Graphene and Polyethylene: A Strong Combination towards Multifunctional Nanocomposites. Polymers.

[57] Flores A., Calleja F.J.B. (1998). Mechanical Properties of Poly(Ethylene Terephthalate) at the near Surface from Depth-Sensing Experiments. Philos. Mag. A.

[58] Chen J., Guo X., Tang Q., Zhuang C., Liu J., Wu S., Beake B.D. (2013). Nanomechanical Properties of Graphene on Poly(Ethylene Terephthalate) Substrate. Carbon N. Y..

[59] Pang H., Xu L., Yan D.X., Li Z.M. (2014). Conductive Polymer Composites with Segregated Structures. Prog. Polym. Sci..

[60] Lu C., Chen X. (2019). Electrospun Polyaniline Nanofiber Networks toward High-Performance Flexible Supercapacitors. Adv. Mater. Technol..

[61] Li S., Zheng G., Wang X., Chen Y., Wu D., Sun D. Improved Electrical Conductivity of PANI/PEO Polymer via Electrospinning and Its Application as NH3 Gas Sensor. Proceedings of the 8th Annual IEEE International Conference on Nano/Micro Engineered and Molecular Systems.

[62] Simotwo S.K., Delre C., Kalra V. (2016). Supercapacitor Electrodes Based on High-Purity Electrospun Polyaniline and Polyaniline-Carbon Nanotube Nanofibers. ACS Appl. Mater. Interfaces.

[63] Malakhova Y.N., Korovin A.N., Lapkin D.A., Malakhov S.N., Shcherban V.V., Pichkur E.B., Yakunin S.N., Demin V.A., Chvalun S.N., Erokhin V. (2017). Planar and 3D Fibrous Polyaniline-Based Materials for Memristive Elements. Soft Matter.

[64] Liu W., Zhong T., Liu T., Zhang J., Liu H. (2020). Preparation and Characterization of Electrospun Conductive Janus Nanofibers with Polyaniline. ACS Appl. Polym. Mater..

[65] Chen C.Y., Huang S.Y., Wan H.Y., Chen Y.T., Yu S.K., Wu H.C., Yang T.I. (2020). Electrospun Hydrophobic Polyaniline/Silk Fibroin Electrochromic Nanofibers with Low Electrical Resistance. Polymers.

[66] Fotia A., Malara A., Paone E., Bonaccorsi L., Frontera P., Serrano G., Caneschi A. (2021). Self Standing Mats of Blended Polyaniline Produced by Electrospinning. Nanomaterials.

[67] Bednarczyk K., Matysiak W., Tański T., Janeczek H., Schab-Balcerzak E., Libera M. (2021). Effect of Polyaniline Content and Protonating Dopants on Electroconductive Composites. Sci. Rep..

[68] Raeesi F., Nouri M., Haghi A.K. (2009). Electrospinning of Polyaniline-Polyacrylonitrile Blend Nanofibers. e-Polymers.

[69] Karbownik I., Rac-Rumijowska O., Fiedot-Toboła M., Rybicki T., Teterycz H. (2019). The Preparation and Characterization of Polyacrylonitrile-Polyaniline (PAN/PANI) Fibers. Materials.

[70] Qu C., Zhao P., Wu C., Zhuang Y., Liu J., Li W., Liu Z., Liu J. (2021). Electrospun PAN/PANI Fiber Film with Abundant Active Sites for Ultrasensitive Trimethylamine Detection. Sens. Actuators B Chem..

[71] Kutanis S., Karakışla M., Akbulut U., Saçak M. (2007). The Conductive Polyaniline/Poly(Ethylene Terephthalate) Composite Fabrics. Compos. Part A Appl. Sci. Manuf..

[72] Gorrasi G., Bugatti V., Milone C., Mastronardo E., Piperopoulos E., Iemmo L., Di Bartolomeo A. (2018). Effect of Temperature and Morphology on the Electrical Properties of PET/Conductive Nanofillers Composites. Compos. Part B Eng..

[73] Xu Q., Wang C., Wang B., Chen Y., Wang H. (2017). In Situ Polymerization and Characterization of Graphite Nanoplatelet/Poly(Ethylene Terephthalate) Nanocomposites for Construction of Melt-Spun Fibers. RSC Adv..

